# Effect of Reaction Temperature on the Microstructure and Properties of Magnesium Phosphate Chemical Conversion Coatings on Titanium

**DOI:** 10.3390/molecules28114495

**Published:** 2023-06-01

**Authors:** Yi-Bo Li, Yu-Peng Lu, Chun-Miao Du, Kang-Qing Zuo, Yu-Ying Wang, Kang-Le Tang, Gui-Yong Xiao

**Affiliations:** 1 Key Laboratory for Liquid-Solid Structural Evolution and Processing of Materials, Ministry of Education, Shandong University, Jinan 250061, China; 2 School of Materials Science and Engineering, Shandong University, Jinan 250061, China

**Keywords:** newberyite, phosphate chemical conversion, reaction temperature, titanium

## Abstract

Magnesium phosphate (MgP) has garnered growing interest in hard tissue replacement processes due to having similar biological characteristics to calcium phosphate (CaP). In this study, an MgP coating with the newberyite (MgHPO_4_·3H_2_O) was prepared on the surface of pure titanium (Ti) using the phosphate chemical conversion (PCC) method. The influence of reaction temperature on the phase composition, microstructure, and properties of coatings was systematically researched with the use of an X-ray diffractometer (XRD), a scanning electron microscope (SEM), a laser scanning confocal microscope (LSCM), a contact angle goniometer, and a tensile testing machine. The formation mechanism of MgP coating on Ti was also explored. In addition, the corrosion resistance of the coatings on Ti was researched by assessing the electrochemical behavior in 0.9% NaCl solution using an electrochemical workstation. The results showed that temperature did not obviously affect the phase composition of the MgP coatings, but affected the growth and nucleation of newberyite crystals. In addition, an increase in reaction temperature had a great impact on properties including surface roughness, thickness, bonding strength, and corrosion resistance. Higher reaction temperatures resulted in more continuous MgP, larger grain size, higher density, and better corrosion resistance.

## 1. Introduction

Among all medical metal materials, Ti and its alloys are most widely used as standard materials for implants in dental and orthopedic contexts [1,2]. Although Ti exhibits excellent biocompatibility and corrosion resistance, it is still an abiotic component and may cause a soft foreign body reaction after implantation and ultimately lead to implantation failure [1]. Limited bioactivity increases the risk of infection in the peri-implant tissue and restricts osteoconduction and osseointegration between Ti and living bone [3,4].

To solve the above outstanding problems, a great many coating preparation techniques have been developed in the field of Ti surface modification [5,6]; methods such as wet chemical methods, electrochemically assisted deposition, and physical vapor deposition are common [7,8,9]. However, these methods still exhibit some limitations because of complex operation, high cost, and the presence of toxic substances [10,11]. Phosphate chemical conversion (PCC), a traditional and well-established metal pretreatment technique, has been introduced into the biomedical field for the purpose of improving the surface properties of metal implants [12].In addition, the in situ growth characteristics of phosphate crystals in the PCC process causes the coating to have good adhesion to the substrate [13]. In surface modification, the bonding strength of the coating is a critical aspect of metal implants [14]. Coatings with poor bonding strength can be easily separated from the substrates, reducing the long-term stability of the implants [15]. According to previous research, the reaction temperature is an influential process parameter in the formation of phosphate coatings [16]. When the temperature is too low, the crystal cannot form a nucleus due to insufficient reaction kinetics. On the contrary, if the temperature is too high, the sediment at the bottom of the solution increases, resulting in low-phosphate-solution consumption [17,18,19]. Moreover, variation in the reaction temperature affects the size and morphology of the crystals, which can cause changes in the thickness and bonding strength of the PCC coating [20,21]. Differences in the compactness and integrity of phosphate coating can impact the corrosion resistance of the coating [19,22,23]. Roughness and wettability, important properties of the surface, can also be affected by changes in the coating microstructure [24].

In recent years, phosphates of magnesium (Mg), calcium (Ca), strontium (Sr), and zinc (Zn) have drawn increased attention in the biomedical field [13,25,26,27]. Among them, MgP is a novel type of bioactive coating that has significant application prospects. MgP not only has minimal cytotoxicity but also has good biocompatibility with osteoblasts, as well as biological characteristics comparable to CaP bone replacements [28,29,30]. Additionally, MgP biomaterial has good biodegradability. Its degradation products, Mg^2+^ and PO_4_^3−^, are essential nutrients for human body that can take part in normal metabolism and keep the body in balance [30,31]. MgP actually includes struvite (MgNH_4_PO_4_·6H_2_O), newberyite (MgHPO_4_·3H_2_O), and cattiite (Mg_3_(PO_4_)_2_·22H_2_O), all of which occur naturally in physiologically and pathologically mineralized tissues [30]. M. Meininger produced a Sr-doped struvite coating on a Ti surface using electrochemically assisted deposition [32]. Suzette Ibasco et al. prepared a struvite coating on a Ti surface using the sputtering method, which increased osteoblast adhesion and cell viability [33]. However, the presence of ammonium salts in struvite may impair the biocompatibility of the material and the release of toxic ammonia or ammonium ions during processing and storage, limiting their use in biomedical applications [34]. As a MgP phase, newberyite has similar properties to CaP (e.g., hydroxyapatite and phosphochalcite) and can encourage osteoblast differentiation [35,36]. Currently, MgP coatings based on the newberyite phase have been studied extensively on the surfaces of magnesium- and iron-based metals, whereas little attention has been paid to the surface modification of Ti [17,35,37,38,39]. Prabaha Sikder used the microwave irradiation technique to create Ag-doped newberyite coatings with antibacterial properties and good cytocompatibility [28]. However, there is still a lack of research investigating the effect of temperature on the generation of newberyite coatings on Ti substrates and the mechanism of its formation via the PCC method.

Therefore, in this study, MgP coatings with the newberyite were fabricated on Ti substrates using the PCC method, and the influence of reaction temperature on their phase composition, microstructure, and properties was comprehensively investigated. Meanwhile, the relevant formation mechanism was also elaborated in detail.

## 2. Results and Discussion

### 2.1. Phase Composition

**Figure 1:**
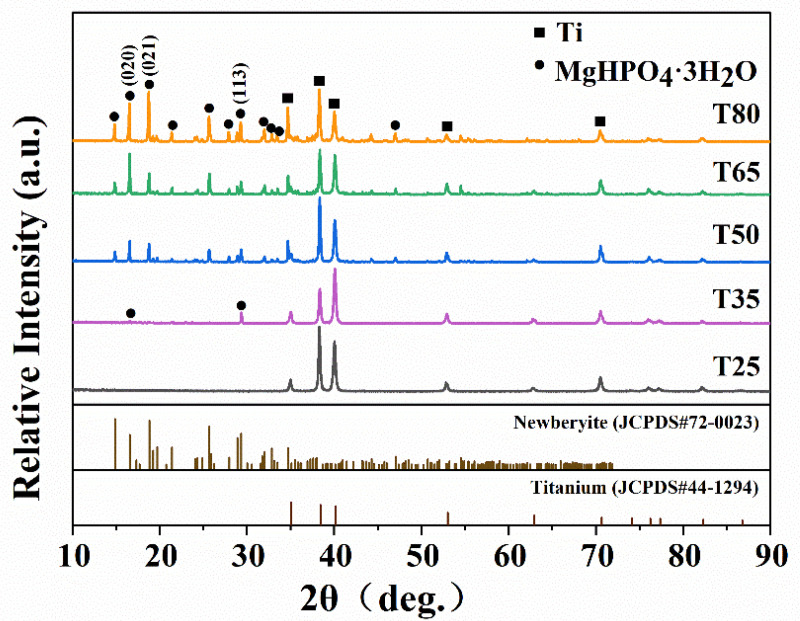
XRD patterns of the coatings on Ti substrate treated at different reaction temperatures (from 25 to 80 °C).

The XRD patterns of the samples at different reaction temperatures are shown in Figure 1. Newberyite (MgHPO_4_·3H_2_O) has an orthorhombic crystal structure, that belongs to the Pbca space group with lattice constants of a = 1.0203 nm, b = 1.067 nm, c = 1.0015 nm, and Z = 8. The results show that the T25 sample is consistent with the bare Ti phase, indicating that the nucleation growth of MgP crystals on Ti is unsuitable at room temperature. In addition, the T35 sample presents weak peaks at 16.58° and 29.34°, corresponding to (020) and (113) crystal planes, respectively. However, high and strong diffraction peaks are detected on the T65 and T80 samples. Additionally, the strongest diffraction peaks are observed at 80 °C, with the (021) plane dominating the coating. Under different reaction temperatures, the relative growth rate of different crystal planes varies, affecting the growth morphology that is manifested by the alteration of the diffraction intensity of crystal planes [40]. Higher temperatures result in larger sizes and higher levels of crystallinity of the crystals. The results are consistent with the report by Zhou et al., affirming that the growth direction of MgP crystals can be affected by temperature [18].

### 2.2. Microstructure of the Coatings

**Figure 2:**
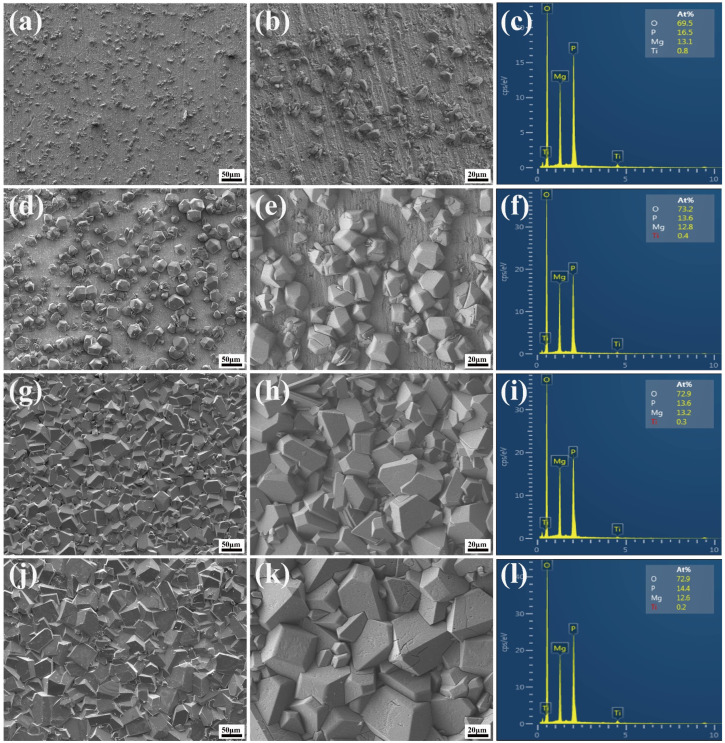
FE-SEM micrographs and elemental compositions of the coatings on Ti substrate treated at different reaction temperatures. (**a**–**c**) T35; (**d**–**f**) T50; (**g**–**i**) T65; (**j**–**l**) T80.

Figure 2 displays the morphologies and elemental compositions of the MgP coatings on the Ti substrate at various reaction temperatures. The results of XRD analysis revealed that no newberyite crystals are observed at 25 °C, indicating inadequate reaction kinetics for nucleation growth at this point. However, at 35 °C, a limited number of crystals start to nucleate and sporadically distribute on the Ti substrate, as depicted in Figure 2a. With temperature increasing to 50 °C, the number and size of grains increase relatively. Nevertheless, no continuous coating is formed. At 65 °C, the Ti surface is completely coated, but the coating is not uniform and is made up of cross-stacked MgP crystals, exhibiting similar shapes but various sizes. Due to the height disparity, there are substantial spaces between large and small grains. Notably, the grain size increases drastically when the temperature increases. The formation of newberyite is a heat-absorbing reaction [17]. The reaction kinetics are complete at 80 °C, allowing the crystals to develop fully and grow up to 70 μm in the deposited MgP crystals. Due to the large particle size and good crystallization of the crystals, the associated diffraction peaks likewise have the highest intensity. The coating is initially created by the impact aggregation of large crystals, followed by the secondary nucleation of small crystals around the corners and edges of the large crystals, resulting in the formation of a denser and more uniform coating. These results demonstrate that the reaction temperature can affect the kinetics of crystal formation.

**Figure 3:**
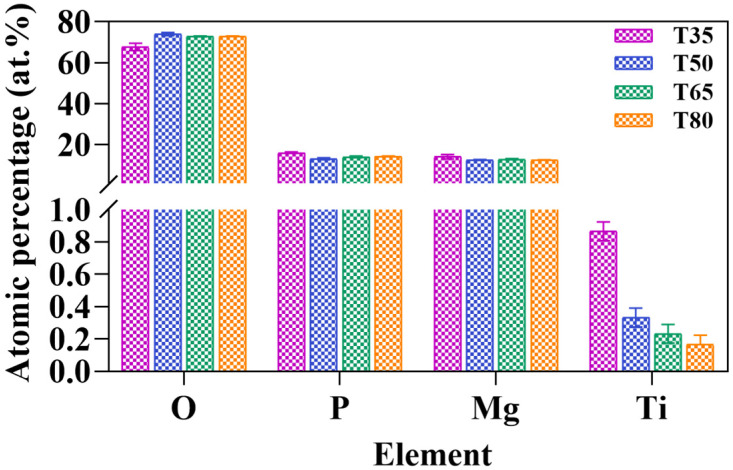
The mean and standard deviation of the elemental content of the samples tested by EDS.

The EDS test revealed the presence of O, P, and Mg elements in samples treated at different temperatures. Figure 3 shows the mean and standard deviation values of the elemental content of the samples tested by EDS. It is worth noting that the atomic ratios of all samples are nearly 7:1:1, which is consistent with the atomic ratio of MgHPO_4_·3H_2_O discovered through XRD analysis.

### 2.3. Corrosion Characteristics

**Figure 4:**
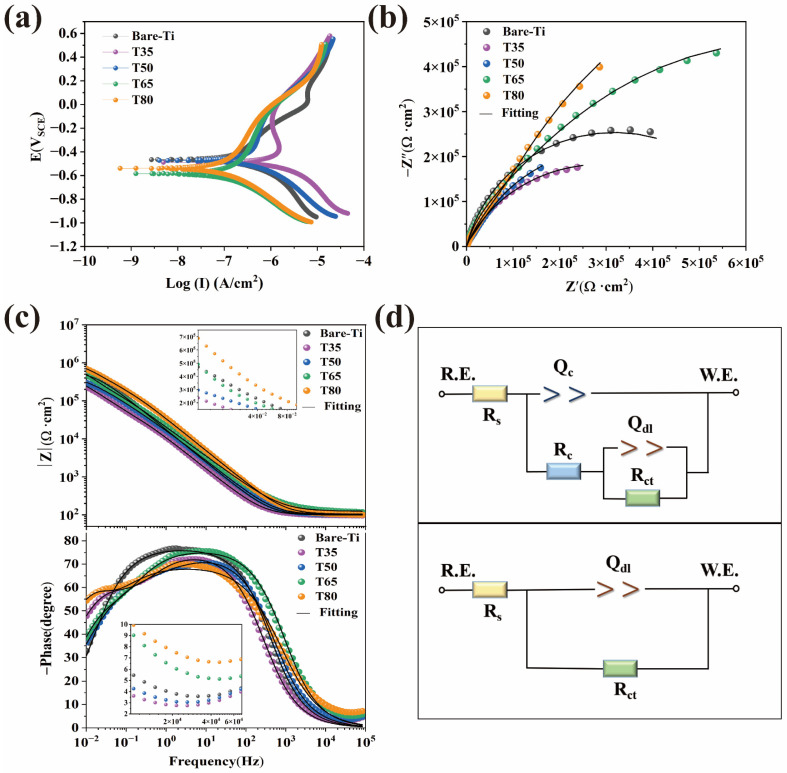
The electrochemical performance tests of the bare Ti and the coatings treated at different reaction temperatures. (**a**) Potentiodynamic polarization curves, and impedance spectra presented in (**b**) Nyquist plots, (**c**) bode amplitude and phase–angle plots, and (**d**) equivalent electrical circuits used to fit the impedance behaviors. R.E.: reference electrode; W.E.: working electrode.

Figure 4a shows the potentiodynamic polarization (PDP) curves of bare Ti and coated samples tested in 0.9% NaCl solution after being prepared at different reaction temperatures. The various electrochemical corrosion parameters, determined by extrapolating the Tafel polarization curves, are listed in Table 1. The corrosion potential (E_corr_) and corrosion current density (I_corr_) can be utilized to assess the level of protection of the samples. Generally, the higher the E_corr_ and the lower the I_corr_ are, the better the corrosion resistance will be [41]. However, the I_corr_ remains the most important factor to consider [19]. Due to the formation of a natural oxide (TiO_2_) coating, the Ti surface exhibits a clear anodic passivation zone on the polarization curve [42]. Bare Ti shows the stronger E_corr_ (−0.466 V) and lower I_corr_ (62.50 × 10^−8^ A·cm^2^) values after a brief immersion in 0.9% NaCl solution. As shown, the I_corr_ of the T65 and T80 samples change direction to a lesser extent compared with bare Ti. The T80 sample has the lowest corrosion current density (26.36 × 10^−8^ A·cm^2^). Additionally, it can be deduced from Table 1, the polarization resistance (R_p_) value of T65 and T80 samples is one order of magnitude greater than that of bare Ti. The variation of R_p_ values for different samples is consistent with that of I_corr_. These results suggest that the T65 and T80 coatings produced at high temperatures offer effective corrosion protection for the bare Ti.

The previous PDP research only provides a qualitative evaluation of the effectiveness of the coating as a protective layer [40]. To thoroughly examine the impact of various temperatures on the electrochemical behavior of the MgP coatings in corrosive fluids, electrochemical impedance spectroscopy (EIS) tests and related equivalent electrical circuits (EEC) simulations are carried out. The Nyquist plots are shown in Figure 4b, where every sample examined is used to plot a curve with a portion of a semicircular arc. In addition, the size of the capacitance ring diameter reflects the corrosion resistance of the coating. Compared with that of bare Ti, the arc diameters of the samples with fully coated T65 and T80 are larger, whereas those of the discontinuously coated T35 and T50 are smaller. The T80 coating stands out among them for having the largest circular diameter and exhibits high anti-polarization behavior, which is compatible with the conclusions drawn from the polarization curve. Figure 4c shows the corresponding bode amplitude and phase angle plots. The corresponding impedance modulus (|Z|) in the low-frequency region (10^−2^ Hz) represents the R_p_, and the phase angle in the high-frequency region (10^5^ Hz) corresponds to the corrosion resistance [43]. Generally, as the temperature rises, |Z| increases in the low-frequency zone and phase angle improves in the high-frequency region. The T80 coating has the highest value of |Z| and the largest phase angle, while the |Z| and phase angle of bare Ti are second only to those of the T65 sample. Since Ti has inherently high corrosion resistance, these analyses further demonstrate the excellent corrosion resistance of the fully coated Ti substrate (T65, T80) with MgP. The results of PDP and EIS also demonstrate the trend of corrosion resistance between the coated samples using various reaction temperatures.

The phase angle–frequency plot in Figure 4c illustrates the difference between the relaxation characteristics of bare Ti and coated samples. The coated samples have two relaxation capacitance constants and bare Ti samples only have one, indicating the presence of two interfaces (solution/coating and coating/substrate) in coated samples [44]. Therefore, in order to suit the EIS results for bare and coated Ti samples, two distinct equivalent electrical circuits (EEC) are applied in this research. Figure 4d displays the EEC adjusted to the impedance results. R_s_, R_ct_, and R_c_ represent the solution, the charge transfer, and the coating resistance, respectively. Q_c_ is the coating capacitance, and Q_dl_ is the electric double-layer capacitance. The ideal capacitance (C) is substituted by the Q constant phase element (CPE) because the non-uniform coating surface causes the electric double-layer capacitance of the electrodes to vary from the ideal capacitance in terms of frequency response characteristics [45]. It is represented by the equations Q = Cωn^−1^, ω = 2πf, where ω is the angular frequency, f is the frequency, and *n* is the fractional index of deviation from the ideal capacitance, reflecting the sample surface uniformity (0 ≤ *n* ≤ 1, *n* = 1 for ideal capacitance) [43]. Table 2 lists the corresponding fitted parameters, where the chi-square parameter (χ^2^) is always less than 0.01, a fact which suggests a good fit and only a minor error between the test spectrum and fitted curve. The findings are consistent with the results of PDP measurements, indicating the high resistance (R_c_ + R_ct_) values of the coatings prepared at high temperatures (T65 and T80) compared to low temperatures (T50 and T35). Therefore, uniform coatings can significantly enhance the corrosion resistance of Ti.

Increasing the temperature generally accelerates crystal growth, causing crystals to pile up to produce a uniform and dense coating. The T35 and T50 samples, created at low temperatures, have no protective effect on the Ti because they fail to form a continuous coating, resulting in a higher I_corr_, more negative E_corr_, and a lower R_p_ value than bare Ti. On the contrary, T65 and T80 samples effectively protect the substrate from defects and corrosion due to the formation of a continuous and tight coating. Particularly for the T80 sample, small crystals continue to grow at the gaps between the large crystals in high-temperature conditions. This process decreases the porosity and increases the density of the coating, providing improved protection for the Ti substrate.

**Table 1:** Electrochemical corrosion parameters determined by potentiodynamic polarization curves of the bare Ti and coated samples treated at temperatures from 35 to 80 °C. Values are shown as mean ± SD, *n* = 3.

Sample	E_corr_ (V)	I_corr_ (×10^−8^ A/cm^2^)	β_a_ (V·dec^−1^)	−β_c_ (V·dec^−1^)	R_p_ (×10^4^ Ω·cm^2^)
Bare Ti	−0.466 ± 0.009	62.50 ± 3.37	0.373 ± 0.007	0.150 ± 0.004	7.430 ± 0.675
T35	−0.489 ± 0.011	468.89 ± 7.42	0.639 ± 0.010	0.173 ± 0.021	1.263 ± 0.022
T50	−0.473 ± 0.014	95.25 ± 3.58	0.399 ± 0.006	0.153 ± 0.017	5.047 ± 0.286
T65	−0.585 ± 0.017	28.22 ± 1.66	0.245 ± 0.001	0.170 ± 0.002	15.43 ± 0.954
T80	−0.540 ± 0.009	21.36 ±1.25	0.259 ± 0.003	0.163 ± 0.004	20.31 ± 0.977

**Table 2:** EIS fitted parameters of the equivalent electrical circuits for bare Ti and coated samples treated at temperatures from 35 to 80 °C. Values are shown as mean ± SD, *n* = 3.

Samples	Bare Ti	T35	T50	T65	T80
R_s_ (Ω⋅cm^2^)	106.6	98.87	102.2	104.5	124.2
Q_dl_ (×10^−5^ Ω^−1^⋅cm^−2^⋅S^−*n*^)	1.114	1.898	1.191	0.6027	1.174
n_dl_	0.9005	0.841	0.8304	0.8504	0.7715
R_ct_ (×10^5^ Ω⋅cm^2^)	3.455	0.7902	0.9703	3.789	5.364
Q_c_ (×10^−5^ Ω^−1^⋅cm^−2^ S^−*n*^)		1.677	0.6252	0.7354	1.066
n_c_		0.7685	0.5822	0.7669	0.9544
R_c_ (×10^5^ Ω⋅cm^2^)		5.292	5.377	9.324	13.11
χ^2^ (10^−3^)	1.56	0.559	5.26	1.14	2.82

### 2.4. Surface Roughness and Wettability

**Figure 5:**
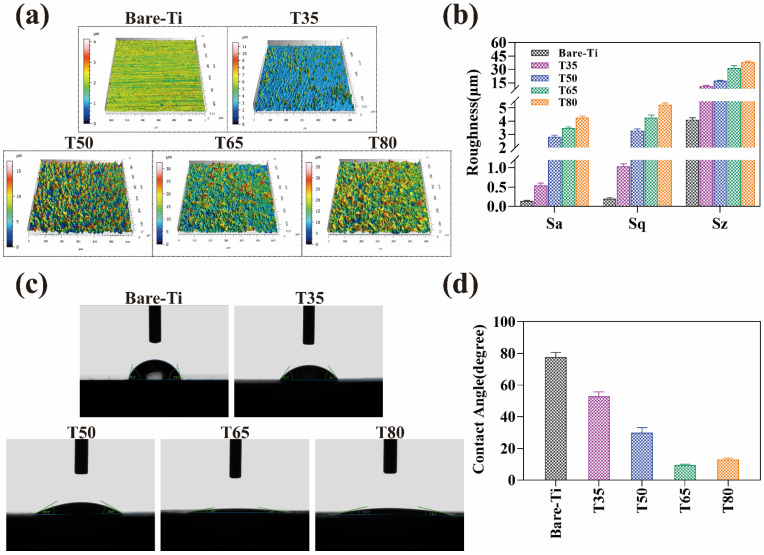
(**a**) LSCM topographical 3D images, (**b**) quantitative measurements of surface roughness, (**c**) digital photos of the water droplets, and (**d**) contact angles (θ) of the bare Ti and the coatings treated at different reaction temperatures.

Surface roughness and wettability are important properties of the material. To further analyze the effect of newberyite on the surface properties of the Ti, roughness and contact angle measurements are performed. The morphological characteristics and roughness values (Sa, Sq, Sz) of the coatings acquired at different temperatures are given in Figure 5a,b. Bare Ti shows parallel scratches after sanding with fine sandpaper, indicating that this typical scratch groove has a low surface roughness and is relatively regular. However, when Ti substrates are submerged in a phosphate solution and heated to various temperatures, crystals of various sizes grow on their surface. Accordingly, irregular peak and valley morphologies are distributed on the 3D topography, resulting in high surface roughness. For the T35 sample, scattered small hill bumps are visible while scratches on the surface of the Ti remain. In contrast, the T50 hills are more closely spaced and have larger peak heights. These morphological features are consistent with their SEM results. The bulges and depressions of the rhombohedral crystals give the T65 and T80 coatings a higher roughness. The 3D topographic map of the T65 sample displays a blue valley around the red peak, indicating that the growth of MgP crystal is uneven and the grain size changes greatly at 65 °C. The T80 coating has the widest range of MgP crystal sizes due to the high temperature, which gives it the largest roughness values. In a word, it can be concluded from the quantitative analysis that the roughness values gradually improve as the temperature increases. This conclusion lines up with the increase in diffraction peak intensity analyzed by XRD above.

Figure 5c,d depict the wettability between different samples through the water droplet morphology and contact angle. There is significant variation in the wettability of bare Ti and MgP coated samples. Water droplets on the bare Ti have a hemispherical shape with a contact angle of 77.70 ± 3.01°. The wettability of T35 and T50 samples significantly increases with the presence of newberyite compared to that of bare Ti. The contact angle of T35 sample decreases to 53.70 ± 2.71° despite the presence of only several scattered grains on its surface. Due to the high hydrophilicity of newberyite, the contact angle of the coating completely covered with crystals is significantly lower than that of the Ti substrate [37]. The wettability improves because the grain size expands as temperature rises, increasing the surface area that can be in touch with water droplets. The contact angle of the T80 sample is as low as 13.13 ± 0.85°. Although T65 and T80 samples have similar wetting angles, SEM analysis shows that T65 samples have non-uniform coating and significant height difference between large and small grains. The gap brought by the height difference lessens the contact angle, resulting in the lowest contact angle measured on the T65 surface, one as low as 9.53 ± 0.61°.

Generally speaking, the corrosion resistance of a material is importantly related to its surface wettability: the more hydrophobic the material surface is, the higher the corrosion resistance will be [46]. However, it has been found that the coatings constructed using newberyite not only have high hydrophilic but also exhibit good corrosion resistance. This is attributed to the continuous and dense newberyite coating prepared on the Ti using the PCC method at a higher temperature, providing good protection to the Ti substrate. On the other hand, it has been proposed that increased surface roughness and improved wettability can promote the osteogenic differentiation of stem cells on Ti [24,47]. In addition, Hotchkiss et al. discovered that increased surface wettability has stronger immunomodulatory effects on the Ti surface than increased roughness [24]. In this study, the use of newberyite coatings (T65, T80) prepared at high temperatures not only increases the surface roughness but also greatly improves the wettability of Ti, which is advantageous for the development of Ti implants in the biomedical field. These results have a certain guiding significance for surface modification.

### 2.5. Coating Thickness and Bonding Strength

**Figure 6:**
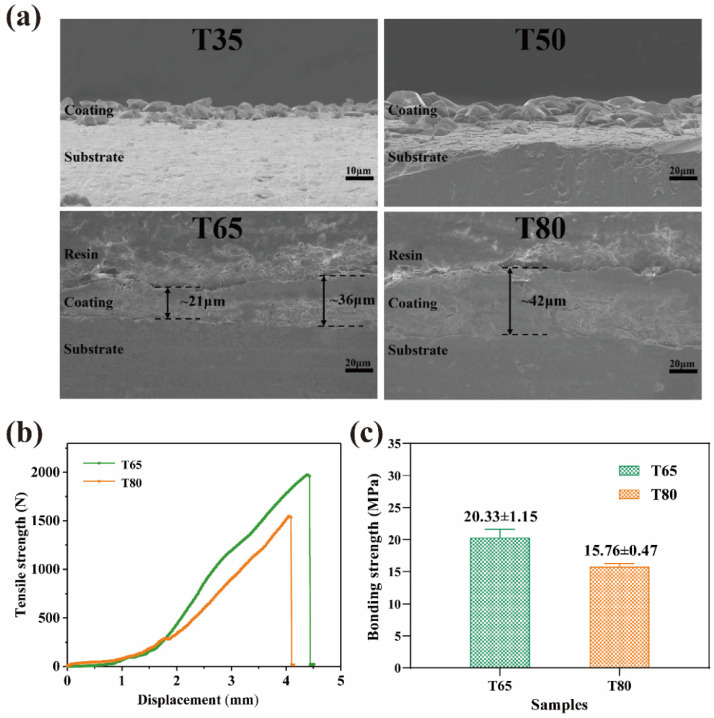
(**a**) Cross-sectional FE-SEM micrographs and thickness of the MgP coatings on Ti substrate, (**b**) tensile–displacement curves, and (**c**) bonding strength variation of different samples.

The bonding strength of the coating is a key feature in surface modification. Figure 6a displays the cross-sectional thickness, morphology, and bonding strength of the samples processed at various reaction temperatures. According to the aforementioned examinations, the T35 and T50 samples do not create a continuous coating and, therefore, do not meet the strict meaning of the coating. As a result, the bonding strength of these two samples is not examined in this investigation. The cross-sectional morphology of the samples matches their surface morphology. As the temperature rises, grain size, crystallinity, and coating thickness all increase. These results are consistent with the observations from SEM and XRD. The cross-sectional thickness of T65 sample varies greatly, ranging from 21 μm to 36 μm. This increase is caused by the wide range of crystal sizes. The T80 coating, on the other hand, has a more consistent thickness of approximately 42 μm, which is proportionate to the size of the crystals [21].

Figure 6b,c compare the tensile adhesion of the T65 and T80 samples. The T65 coating has a bonding strength of approximately 20.33 ± 1.15 MPa and the T80 coating has a lesser bonding strength of 15.76 ± 0.47 MPa. These results are mostly due to the thickness and microstructure of the coating, influenced by temperature variations. The reaction kinetics at 65 °C are more suitable for the growth of newberyite crystals. Many small corrosion pits exist on the surface of Ti after acid etching. This provides ample nucleation sites, allowing the crystal to grow appropriately with a structure that matches well with Ti. In addition, the smaller crystal size and coating thickness of the T65 sample allow for a stronger bonding strength of the coating to the Ti substrate. On the contrary, at a high temperature of 80 °C, the reaction kinetic state is so sufficient that the crystals nucleate rapidly and grow into plate-like crystals with large dimensions that are poorly matched to the Ti structure. Moreover, an increase in coating thickness will lead to increased internal stress and brittleness, making it easy to fracture the coating in layers under external forces, which results in reduced bonding strength.

In general, the high interfacial bonding strength between the MgP coating and the Ti substrate can be explained by the in situ growth properties of insoluble newberyite in the PPC solution [20]. In addition, the increase in coating thickness can enhance the corrosion resistance. At the same time, it makes the bonding strength weak. Therefore, to obtain a coating with moderate thickness, high corrosion resistance, and good bonding strength, it is necessary to further study how to balance these factors.

### 2.6. Formation Mechanism

The PCC chemical conversion is an endothermic reaction that leads to the growth of phosphate crystals on the substrate [48]. In addition, this reaction can increase the ionic activity and thermal motion in the PCC solution [17,19]. However, an excessive temperature may lead to large deposits of Mg^2+^ and PO_4_^3−^ at the bottom of the solution, reducing the utilization rate of phosphate solution [40]. Therefore, to examine the effect of reaction temperature on the microstructure and properties of coatings, temperature gradients of 25, 35, 50, 65, and 80 °C are set in this paper.

**Figure 7:**
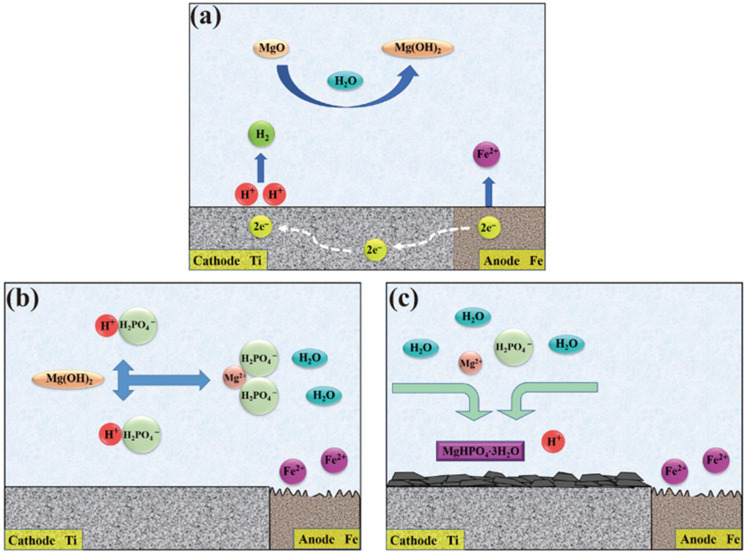
Schematic diagram of the deposition of MgP coating on Ti substrate. (**a**) Hydrogen evolution reaction and Fe corrosion, (**b**) formation of water-soluble Mg(H_2_PO_4_)_2_, and (**c**) newberyite nucleation to form coatings.

The MgP fabricated in this work is obtained from MgO, and the following reaction (Equation (2)) takes place in the solution first. Additionally, Ti/Fe constitutes an electrically coupled system, with Ti serving as the coupling cathode and Fe as the coupling anode according to our previous work [49]. As a result of the potential difference between them, Ti undergoes a cathodic hydrogen precipitation process (Equation (3)) while Fe experiences anodic dissolution (Equation (4)) (Figure 7a). At the substrate–solution interface, the hydrogen precipitation reaction causes a significant increase in pH, which encourages the phosphate ionization reaction (Equation (5)).

(1) $$MgO+H_{2}O\to Mg{\left(OH\right)}_{2}$$

(2) $$2H^{+}+2e^{-}\to H_{2}↑$$

(3) $$Fe-2e^{-}\to {Fe}^{2+}$$

(4) $$H_{2}{{PO}_{4}}^{-}+H^{+}↔H{{PO}_{4}}^{2-}+2H^{+}$$

Under the action of phosphoric acid, the Mg(OH)_2_ produced by Equation (1) transforms into magnesium dihydrogen phosphate (Equation (6)) that is fully dissolved in water (Figure 7b). Finally, when the solution achieves supersaturation, a reaction (Equation (7)) takes place. Besides, insoluble newberyite nuclei begin to deposit on the connected anodic Ti substrate with lower electrical potential, driven by the potential difference in the electrically coupled system (Figure 7c).

(5) $$Mg{\left(OH\right)}_{2}+{2H}_{3}{PO}_{4}\to Mg{\left(H_{2}{PO}_{4}\right)}_{2}+2H_{2}O$$

(6) $${Mg}^{2+}+{H_{2}{PO}_{4}}^{-}+3H_{2}O\to MgHPO_{4}\cdot 3H_{2}O+H^{+}$$

The newberyite phase is unaffected by the reaction temperature; instead, it only affects the quantity and size of crystals. MgP crystals cannot form at 25 °C because the reaction kinetics required to overcome the activation potential of the precipitation process are insufficient. Although raising the temperature by 10 °C in a way that the the kinetic and thermodynamic requirements are satisfied, there are few activated molecules and little phosphate ionization in the solution, resulting in the formation of only several small crystals. Once the temperature reaches 50 °C, a large number of MgP crystals begin to form but do not form a continuous coating. As the temperature rises to 65 °C, the number of active spots on the Ti grows and the supersaturation of the solution likewise rises. Under the control of kinetic factors and thermodynamic driving forces, the crystals are tightly aligned to produce a continuous and dense coating, but the grain growth is not uniform, and the grain size varies. Once the temperature reaches 80 °C, crystals grow uncontrollably and quickly, increasing in size while still having many crystal nuclei, thus allowing for the formation of a closely packed coating with large grains. Additionally, the activation of the substrate in the acid and the surface adjustment in the colloidal titanium solution allow the adsorption of colloidal titanium particles to provide more “active centers” for the reaction and increase the crystal nucleation sites [19]. The local high temperature and high-pressure microenvironment, along with the potent shock waves produced by the ultrasonic field, speed up ions diffusion and collision in the solution, which is favorable for the deposition and coating formation of crystals [50,51].

## 3. Materials and Methods

### 3.1. Coating Preparation

Commercially pure Ti plates (Grade TA2) were cut into square pieces with dimensions of 10 × 10 × 2 mm and used as substrates. All Ti surfaces were sanded with #180, #600, and #1000 silicon carbide abrasive paper in sequence; then, they were put in alcohol and deionized water in turn for the purpose of ultrasonic cleaning in order to obtain a clean surface.

The Ti pieces were clamped with pure iron (Fe) to form an electrically coupled system (Ti/Fe). This was activated by pickling in 2 wt% hydrofluoric acid (HF) solution for 15 s at room temperature, rinsed with deionized water, and then soaked into 3 g/L colloidal Ti solution for 30 s for surface adjustment. This was followed by the immediate immersion in PCC solutions of different temperatures from 25 to 80 °C for 25 min with an ultrasonic reaction. The PCC solution composition and transformation process conditions were shown in Table 3. The pH of the solution was adjusted to 4.2 by sodium hydroxide (NaOH, 5 mol/L) or phosphoric acid (H_3_PO_4_, 7% *v*/*v*). Finally, the obtained samples were cleaned with deionized water and dried in a drying oven at 40 °C. The coated samples were labeled with the phosphating temperature, such as T35, which denotes treatment at 35 °C.

**Table 3:** Chemical compositions of the phosphating solution and treatment conditions.

Bath Composition	Concentration	Treatment Conditions
MgO	10 g/L	PH = 4.2, t = 25 min, T = 35 °C; 50 °C; 65 °C; 80 °C
H_3_PO_4_	20 mL	
HNO_3_	18 mL	
NaNO_2_	2 g/L	
NaNO_3_	2 g/L	
NaOH	2.5 g/L	

### 3.2. Coating Characterization

The microscopic morphology and the elemental composition of the coating were observed and analyzed by a field emission scanning electron microscope (FE-SEM, SU-70, Hitachi, Tokyo, Japan) at 5 kV and 15 kV, respectively. Three regions of a sample were randomly selected for energy dispersive spectrometer (EDS). The phase composition of the coated crystals was examined using an X-ray diffractometer (XRD, DMAX-2500PC, Rigaku, Japan) with Cu-Kα radiation. Under 40 kV and 100 mA control, the scanning angle 2θ was between 10° and 90° and the scanning speed was 4° min^−1^.

A 600 × 600 μm area of the sample surface was measured under the action of a laser confocal scanning microscope (LSCM 800, Zeiss, Oberkochen, Germany). From this, the three-dimensional (3D) morphology of the coating, the average surface roughness (Sa), the root means square roughness (Sq), and the maximum height (Sz) were derived. Surface wettability was measured using a contact angle goniometer (DSA100S, KRUSS, Hamburg, Germany) with a fixed droplet of water (2 μL) on the coating surface, and the contact angle θ value was recorded for 30 s at rest. To make the experimental results accurate, three measurements were randomly selected for each sample.

### 3.3. Tensile Adhesion Tests

In accordance with ASTM C633-01, the bonding strength of the coating was examined using a microcomputer-controlled universal testing device (WDW-5, STAR, Jinan, China) with a maximum test force of 5 kN. Prior to the test, it was necessary to use an acrylic adhesive to bond the coated sample between two stainless steel cylinders whose axes coincided, after which they were cured for 24 h at room temperature. The sample was subjected to a vertical tensile force during the test at a constant rate of 1 mm/min until the specimen was torn away from the bonded surface. The bonding strength of the coating was calculated by dividing the maximum obtained tensile load by the surface area of the samples. Seven parallel samples were set for each sample group, and the average bonding strength was calculated from five stable data.

### 3.4. Electrochemical Measurements

The electrochemical behaviors of the samples were detected using an electrochemical workstation (CHI660e, Shanghai, China) containing a three-electrode cell. In this three-electrode cell system, the saturated calomel electrode (SCE) was the reference electrode, the platinum (Pt) sheet was the counter electrode, and the bare or coated Ti plate was used as the working electrode. A 0.9 wt% NaCl solution at 25 °C was utilized as the electrolyte.

After the open circuit potential (OCP) stabilized for 1800 s, the values were recorded and then the electrochemical impedance spectrum (EIS) was measured. The frequency range and sinusoidal perturbation voltages were from 10^5^ Hz to 10^−2^ Hz and 5 mV, respectively. Finally, Tafel polarization curve was tested at a constant voltage scan rate of 1 mv/s over a potential range of ±0.5 V relative to the OCP. Based on the extrapolation of the Tafel polarization curve, the equilibrium potential (E_corr_), corrosion current (I_corr_) and anode/cathode Tafel slope (*β_a_*/*β_c_*) can be deduced. In addition, polarization resistance (*R_p_*) was calculated according to the following Equation (7). The EIS results were fitted to the equivalent circuit using ZsimpWin software. Triplicate experiments were performed for electrochemical tests.

(7) $$R_{P}=\frac{\beta_{a}\cdot |\beta_{c}|}{2.303\cdot I_{corr}\cdot (\beta_{a}+\left|\beta_{c}\right|)}$$

## 4. Conclusions

In this study, MgP coatings with newberyite of (MgHPO_4_·3H_2_O) were constructed on Ti substrates using the controllable PCC method. The effect of reaction temperature on the phase composition, microstructure, and properties of the coatings was investigated. The results show that the reaction temperature has no obvious effect on the phase composition of the MgP coating. However, the temperature change has a significant effect on the nucleation and growth of newberyite, as well as on the microstructure and properties of the coating. At the same time, the Ti surface transforms from a fragmented and sparse discontinuous coating to a tightly arranged dense coating with the increasing reaction temperature. Moreover, the uniform coatings prepared at 65 °C have better hydrophilicity (9.53 ± 0.61°) and bonding strength (20.33 ± 1.15 MPa) than those prepared at 80 °C. The improvements are mainly related to the changes in the microstructure and thickness of the coatings caused by the temperature variation. In addition, the MgP coating prepared at 80 °C exhibits the maximum thickness (42 μm) and superior corrosion resistance because it has the best uniformity. These results provide essential guidance for developing MgP coating on biomedical metal surfaces. In future work, it is necessary to further investigate the effect of various MgP coatings on their biological characteristics.

## Data Availability

The data presented in this study are available on request from the corresponding author. The data are not publicly available due to issues related to the proprietary rights.
